# Dietary emulsifier consumption accelerates type 1 diabetes development in NOD mice

**DOI:** 10.1038/s41522-023-00475-4

**Published:** 2024-01-06

**Authors:** Clara Delaroque, Benoit Chassaing

**Affiliations:** INSERM U1016, team “Mucosal microbiota in chronic inflammatory diseases”, CNRS UMR 8104, Université Paris Cité, Paris, France

**Keywords:** Microbiome, Microbiota

## Abstract

The rapidly increasing prevalence of type 1 diabetes (T1D) underscores the role of environmental (i.e. non-genetic) determinants of T1D development. Such factors include industrialized diets as well as the intestinal microbiota with which they interact. One component of industrialized diets that deleteriously impact gut microbiota is dietary emulsifiers, which perturb intestinal microbiota to encroach upon their host promoting chronic low-grade intestinal inflammation and metabolic syndrome. Hence, we investigated whether 2 dietary emulsifiers, carboxymethylcellulose (CMC) and polysorbate-80 (P80), might influence the development of T1D in NOD mice, which spontaneously develop this disorder. We observed that chronic emulsifier exposure accelerated T1D development in NOD mice, which was associated with increased insulin autoantibody levels. Such accelerated T1D development was accompanied by compositional and functional alterations of the intestinal microbiota as well as low-grade intestinal inflammation. Moreover, machine learning found that the severity of emulsifier-induced microbiota disruption had partial power to predict subsequent disease development, suggesting that complex interactions occur between the host, dietary factors, and the intestinal microbiota. Thus, perturbation of host–microbiota homeostasis by dietary emulsifiers may have contributed to the post-mid-20th-century increase in T1D.

## Introduction

Type 1 diabetes (T1D) is caused by the autoimmune destruction of pancreatic β cells in genetically predisposed individuals, resulting in severe insulin deficiency and hyperglycemia requiring life-lasting treatment with exogenous insulin. With a prevalence reaching one in 250 people^1^, T1D is one of the most prevalent autoimmune diseases in children. T1D results from an individual-specific and complex interplay between environmental factors, microbiome, genome, metabolism, and immune system^1,2^. While it is well established that genetics play a central role in T1D, with both HLA and non-HLA associated loci characterized^3^, understanding environmental factors that influence T1D development is poorly understood, although recent findings suggest a role for gut microbes. For example, the ‘The Environmental Determinants of Diabetes in the Young’ (TEDDY) study demonstrated a link between T1D and enterovirus infection^4^, early life metabolism^5^, and specific microbiota signature^6–8^. The role of the gut microbiota in T1D development has been demonstrated in both humans^9,10^ and mouse models^11–13^, wherein it is a key factor in the mechanisms governing T1D development.

A large array of factors can impact the intestinal microbiota, both at the compositional and functional levels, leading to dysbiosis. For example, we previously reported that dietary emulsifiers, additives commonly added in ultra-processed food, are detrimentally impacting the intestinal microbiota, in both mice and human^14–16^. More specifically, dietary emulsifier consumption alters microbiota composition and gene expression in a way that increases its potential to promote inflammation. Furthermore, emulsifier consumption results in select microbiota members encroaching upon the host by penetrating the normally sterile inner mucus layer^14–16^. Importantly, these alterations are necessary and sufficient to drive chronic intestinal inflammation and downstream detrimental consequences such as metabolic deregulations and increased colitis susceptibility^14–16^. Ultra-processed food in general, and emulsifiers in particular, has been increasingly consumed worldwide by both adults and children over the past few decades^17^. This observation aligns with T1D incidence and prevalence overall annual increases of about 2–3% per year^1^. In addition, Northern America and Europe being top positions worldwide for the annual number of T1D cases^1,18^ correlates with the observation for these regions to also be high emulsifier consumers^19^. Hence, we investigated here whether dietary emulsifier consumption could impact T1D development in mice, and the role played by the intestinal microbiota in such phenomena.

In the present study, we treated non-obese diabetic (NOD) mice, a model of spontaneous T1D^20^, with dietary emulsifier carboxymethylcellulose (CMC) or polysorbate-80 (P80) from the age of 4 weeks. We observed that CMC consumption accelerates T1D development with an increased level of circulating insulin autoantibody as soon as 4 weeks of exposition. Emulsifiers-treated mice presented low-grade intestinal inflammation in a way that positively correlated with the rapidity of diabetes establishment. Moreover, emulsifier consumption resulted in stark microbiota alterations in terms of composition, richness, localization, and pro-inflammatory potential. Interestingly, microbiota compositional and functional quantitative features were not sufficient to reliably predict T1D development, suggesting that complex interactions occur between the host, dietary factors, and the intestinal microbiota to promote T1D.

## Results

### Dietary emulsifier consumption accelerated type 1 diabetes development in NOD mice

**Fig. 1:**
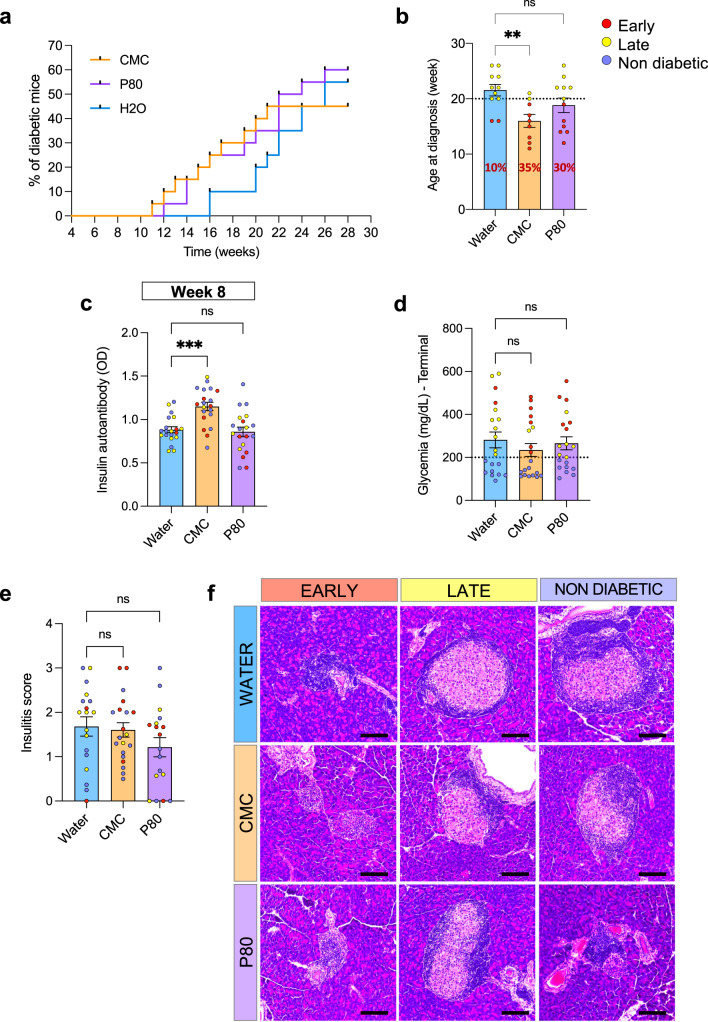
Dietary emulsifier consumption accelerates type-1 diabetes development in NOD mice. NOD mice were exposed to CMC or P80 (1%) in the drinking water for 28 weeks and diabetes development was evaluated weekly through blood glucose measurements. **a** Incidence of type-1 diabetes according to time. **b** Age at diagnosis, with a bold dark red percentage indicating the proportion of early diagnosed diabetic mice for each group. **c** Insulin auto-antibody levels, measured in serum collected at week 8. **d** Glycemia at diagnosis. **e** Insulitis individual score measured on hematoxylin and eosin-stained pancreatic sections. **f** Representative images of HE-stained pancreatic islets. Scale bar: 100 μm. Data are the means ± s.e.m., and points represent individual mice (*N* = 20). Significance are indicated as **p ≤ 0,01; ***p ≤ 0,001; n.s. indicates nonsignificant.

In order to investigate the effect of emulsifier consumption on T1D development, we exposed NOD mice to 1% CMC or P80 in the drinking water (*n* = 20), beginning at 4 weeks of age and maintained thereafter. Dose was determined based on previous work investigating the effect of various concentrations of CMC and P80 in inducing microbiota alteration and associated low-grade inflammation and metabolic deregulations^14^. Glycemia was measured weekly, and T1D was diagnosed following two glycemia measurements above 200 mg dL^−1^ 72 h apart, as described (Supplementary Fig. 1a). CMC-treated mice exhibited accelerated T1D development compared to water-treated mice, as presented (Fig. 1a, b), with diabetic individuals arising in the CMC group at week 11 of age, whereas the first diabetic individual from the water-treated group was diagnosed at 16 weeks of age (Fig. 1b). A similar trend was observed in P80-treated mice, with the first diabetic mouse diagnosed at the age of 12 weeks, although this latter difference did not reach statistical significance (Fig. 1a, b). Such accelerated T1D development in CMC-treated mice was observed in mice housed in different cages and was accompanied by a precocious increase in serum insulin autoantibody level evident by 4 weeks following the beginning of the treatment (Fig. 1c), with such seropositivity being an early event in T1D development^1^. Next, in order to distinguish mice diagnosed at the beginning of the study from the ones diagnosed later or those that remained diabetic-free, we segregated mice into three groups (early T1D, late T1D, and no T1D) based on the age at diagnosis, as described in Supplementary Fig. 1b^21,22^. This approach revealed the absence of correlations between the precocity of T1D-diagnosis and insulin autoantibody levels. Similarly, while all diabetic mice exhibited glycemia measurements above 200 mg dL^−1^, such levels tended to be higher in the early-diagnosed mice compared to late-diagnosed T1D mice (ANOVA glycemia early T1D vs. late T1D *p* = 0.055), in particular in P80-treated mice (Fig. 1d, Supplementary Fig. 2). Finally, an investigation of pancreatic inflammation revealed that neither dietary emulsifier consumption nor early T1D development was associated with insulitis (Fig. 1e, f). Altogether, these results suggest that commonly used dietary emulsifiers increased the production of insulin autoantibodies and accelerated T1D development, in mice, in a way that appeared to be independent of pancreatic inflammation.

### Gut microbiota composition is altered following dietary emulsifier consumption in NOD mice

**Fig. 2:**
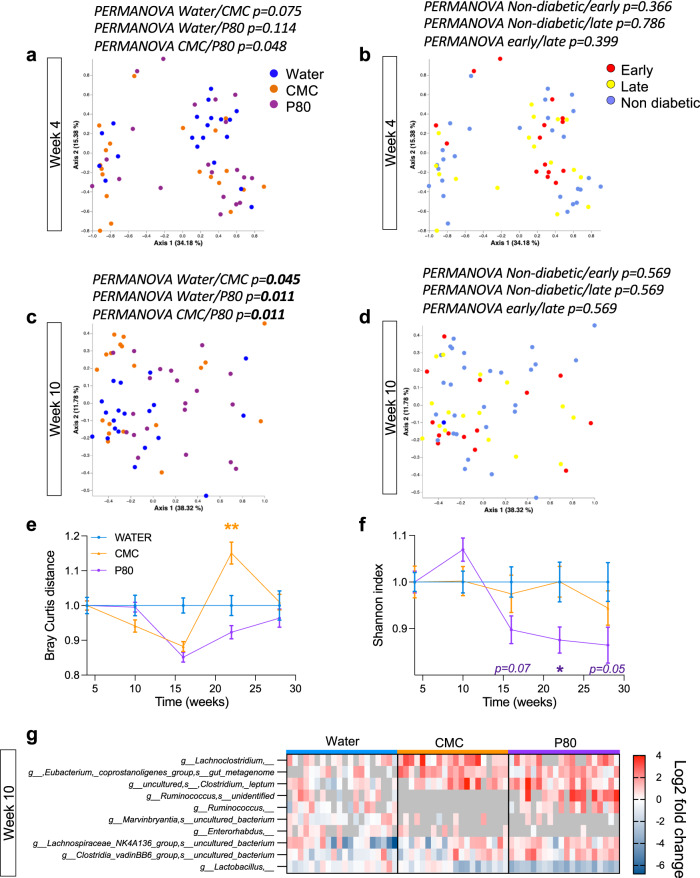
Dietary emulsifier consumption induces alterations in intestinal microbiota composition. NOD mice were exposed to CMC or P80 (1%) in the drinking water for 28 weeks and diabetes development was evaluated weekly through blood glucose measurements. **a** and **b** Principal coordinates analysis of the Bray–Curtis distance at week 4, colored by treatment (**a**) or by diabetes status (**b**). **c** and **d** Principal coordinates analysis of the Bray–Curtis distance at week 10, colored by treatment (**c**) or by diabetes status (**d**). Corrected *p*-values are indicated on the plots. **e** Bray–Curtis distance over time normalized to the water-treated control group. **f** Alpha-diversity Shannon index over time, normalized to the water-treated control group. **g** MaAsLin2-based identification of microbiota members was significantly altered by CMC/P80 treatment at week 10 compared to the water-treated control group. Columns represent individual mice. Data are the means ± s.e.m (*N* = 20). Significance are indicated as **p ≤ 0,01; ***p ≤ 0,001.

Gut microbiota is known to be a determinant of T1D etiology^9,23^ and, furthermore, known to be altered in a deleterious manner by emulsifiers^14^, thus prompting us to analyze how microbiotas of NOD mice were impacted by CMC and P80 via 16S rRNA sequencing. Prior to treatment, microbiota composition did not differ between water, CMC and P80 treated mice (Fig. 2a, PERMANOVA water vs. CMC *p* = 0.075, PERMANOVA water vs. P80 *p* = 0.114), nor between future early/late diabetic and diabetic free mice (Fig. 2b). However, after 6 weeks of treatment, microbiota of both CMC- and P80-treated mice presented compositional alterations, as revealed by significant clustering in Fig. 2c (PERMANOVA water vs. CMC *p* = 0.045, PERMANOVA water vs. P80 *p* = 0.011). Of note, the microbiota composition of CMC-treated mice was significantly different compared to mice exposed to P80 (PERMANOVA CMC vs*.* P80 *p* = 0.011), further suggesting that these two additives differentially act on the intestinal microbiota^16^. Such alteration in microbiota composition was not associated with, and thus not a consequence of, diabetic status (Fig. 2d). Bray–Curtis distance measured between water- and CMC-treated mice was significantly different from the distances measured between individuals within the water group following 18 weeks of treatment (Fig. 2e), while P80 treatment induced a stark decrease in microbiota richness, as indicated by the Shannon diversity index (Fig. 2f). Such observations further support distinct mechanisms underlying CMC- and P80-mediated effect on the intestinal microbiota. Altogether, these observations aligned with previous findings demonstrating that chronic exposure to dietary emulsifiers alters various compositional aspects of the intestinal microbiota. We next performed Microbiome Multivariable Associations with Linear Models (MaAsLin2) analysis to identify microbiota members significantly altered in their abundance following 6 weeks of emulsifier consumption (Fig. 2g). Among the 10 microbiota members identified as being the most significantly impacted, *Lachnoclostridium* was increased in CMC and P80 treated mice, while CMC-consumption induced a specific decrease in *Ruminococcus* (Fig. 2g). Relative abundance of these emulsifiers-affected microbiota members was not correlated with diabetic status, nor with T1D precocity. A similar MaAsLin2 analysis was performed to identify microbiota members whose abundance at week 10 was significantly impacted by diabetes onset, an approach that failed to identify any diabetes-associated microbiota member. Altogether, these results highlight that irrespective of diabetic status, emulsifier consumption led to microbiota alteration in terms of global composition, richness as well as specific microbiota member’s abundance in NOD mice.

### Dietary emulsifier consumption increased microbiota pro-inflammatory potential in NOD mice

**Fig. 3:**
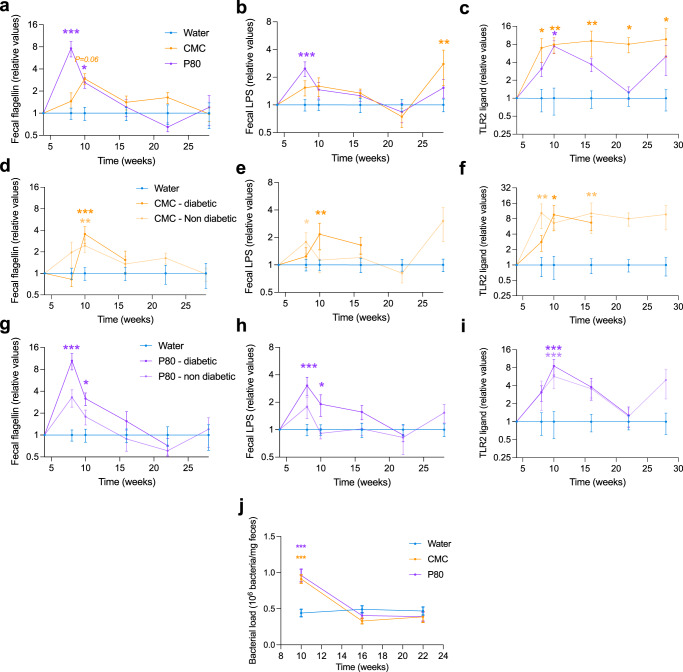
Dietary emulsifier consumption is associated with an increase in the fecal level of microbiota-derived pro-inflammatory molecules. NOD mice were exposed to CMC or P80 (1%) in the drinking water for 28 weeks and diabetes development was evaluated weekly through blood glucose measurements. **a–c** Fecal level of bioactive flagellin (**a**), LPS (**b**), and TLR-2 ligands (**c**), assessed through HEK reporter cells, with data being normalized to water-treated mice and to week 4 (pre-treatment) timepoint. In **e** and **f**, only water- and CMC-treated groups are presented, with CMC-treated mice being separated based on their diabetes status. In **g–i**, only water- and P80-treated groups are presented, with P80-treated mice being separated based on their diabetes status. **j** Fecal bacterial load at weeks 10, 16, and 22 were obtained by flow cytometry approach and expressed as 10^6^ bacteria/mg of fecal material. Data are the means ± s.e.m., and points represent individual mice (*N* = 20). Significance are indicated as *p ≤ 0,05, **p ≤ 0,01; ***p ≤ 0,001.

The potential of the gut microbiota to stimulate the underlying mucosal immune system can be assessed through the quantification of microbiota-derived pro-inflammatory molecules, such as lipopolysaccharide (LPS) and flagellin. Such microbiota-derived molecules have previously been demonstrated as playing a key role in the etiology of T1D^23–25^, and undergo increased abundance following emulsifier consumption^14,26^. Thus, we next measured functional levels of flagellin, LPS, as well as TLR-2 ligands in feces *via* the use of TLR5, TLR4, and TLR2 reporter cells, respectively (Fig. 3a–i). Significant increases in fecal levels of flagellin, LPS, and TLR2-ligands were observed in response to both CMC and P80 by 4 weeks of exposure, indicating a rapid effect of emulsifier exposure on the intestinal microbiota pro-inflammatory potential (Fig. 3a–c). Most of these effects vanished after longer exposures, suggesting a host response dampening microbiota pro-inflammatory potential (Fig. 3a–c)^27,28^. Increase in such pro-inflammatory potential was observed in both diabetic and diabetes-free mice and was not associated with diabetic status (Fig. 3d–i). Cytometry analysis of fecal bacteria suspension obtained from feces collected at weeks 10, 16, and 22 of age revealed a transient two-fold increase in bacterial load in both CMC- and P80-treated mice at week 10 (0.91 × 10^6^ ± 0.26 × 10^6^ and 0.96 × 10^6^ ± 0.39 × 10^6^, respectively), compared to water-treated mice (0.44 × 10^6^ ± 0.23 × 10^6^), as presented Fig. 3j. This suggests that the observed microbiota pro-inflammatory potential increase might be, at least partially, driven by increased bacterial loads. However, dietary emulsifier treatment also induced a 4–16-fold increase in flagellin and TLR2-ligands, suggesting that microbiota composition alteration and/or microbiota transcriptional activity modulation might also contribute to the observed increased microbiota pro-inflammatory potential. Moreover, the increase in fecal TLR ligand levels was not correlated with specific microbiota members (data not shown), further supporting that such an increase is driven by altered microbiota gene expression. Hence, these data altogether suggest the ability of dietary emulsifiers to increase the intestinal microbiota’s pro-inflammatory potential.

### Dietary emulsifier consumption induced microbiota encroachment and increased microbiota targeted antibodies production in NOD mice

**Fig. 4:**
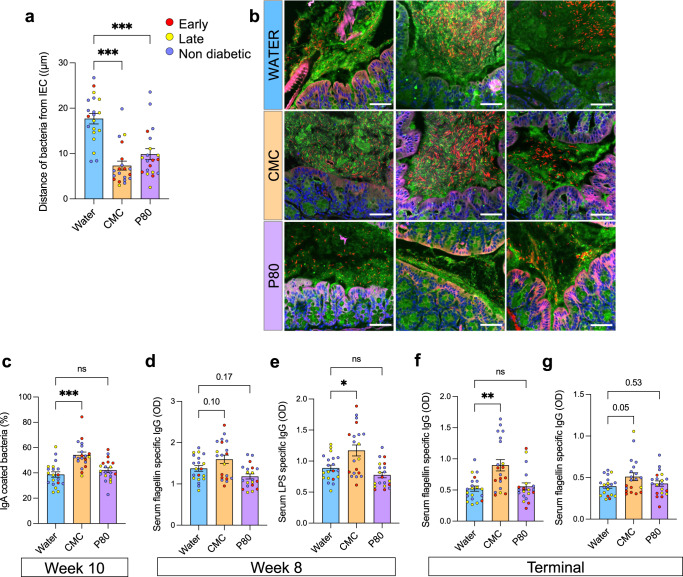
Dietary emulsifiers consumption induces microbiota encroachment and increased circulating level of antibodies targeting microbiota-derived antigens. NOD mice were exposed to CMC or P80 (1%) in the drinking water for 28 weeks and diabetes development was evaluated weekly through blood glucose measurements. **a** Distances of closest bacteria to intestinal epithelial cells (IECs) over five high-powered fields per mouse. **b** Representative confocal microscopy images of microbiota localization: MUC2, green; actin, purple; bacteria, red; and DNA, blue. Scale bar, 50 mm. Pictures are representative of 10 biological replicates. **c** Percentage of IgA-coated fecal bacteria at week 10, assessed by flow cytometry. **d–g** Circulating anti-flagellin (**d** and **f**) and anti-LPS (**e** and **g**) antibodies were measured by ELISA in serum collected at week 8 (**d** and **e**) or at euthanasia (**f** and **g**). Data are the means ± s.e.m, and points represent individual mice (*N* = 20). Significance are indicated as **p ≤ 0,01; ***p ≤ 0,001; n.s. indicates nonsignificant.

The above-described increase in flagellin, LPS, and TLR2 ligands suggests an increased immune-stimulating ability of the intestinal microbiota following dietary emulsifier consumption. Such phenomenon was previously associated with microbiota penetration of the mucus layer characterized by a reduction in the distance separating microbiota members and the intestinal epithelium^14^. Such encroachment is thought to increase the ability of the microbiota to stimulate the underlying immune system, ultimately triggering inflammation. Hence, we next measured such microbiota–epithelium distance, and we observed that CMC- and P80-treated mice exhibited severe microbiota encroachment (Fig. 4a, b), with the average bacteria/epithelium distance being reduced from 17.69 ± 1.16 μm in water-treated mice to 7.33 ± 0.99 μm in CMC- and 9.86 ± 1.20 μm in P80-treated mice. Moreover, such microbiota encroachment was not associated with T1D precocity, suggesting that such an emulsifier-induced phenomenon is not the sole actor beyond T1D promotion induced by these food additives (Supplementary Fig. 3a).

The above-mentioned observations revealed that dietary emulsifier exposure is sufficient to increase intestinal microbiota pro-inflammatory potential as well as to promote microbiota encroachment, suggesting an increased capacity of such emulsifier-shaped microbiota to stimulate the underlying mucosal immune system. Well-aligned with this concept, we observed an increased level of fecal IgA-coated bacteria at week 10 (Fig. 4c), together with an increase in serum levels of anti-flagellin and anti-LPS antibodies in CMC-treated mice (Fig. 4d–g). Here again, T1D precocity was not associated with the highest level of fecal or circulating antibodies level (Fig. 4c–g). Such an increase in luminal and circulating bacteria-targeting IgA was restricted to CMC mice, suggesting that the implementation of such host-mediated defense response is specific to CMC-induced alteration. Hence, dietary emulsifier consumption is sufficient to induce alteration in microbiota composition, richness, pro-inflammatory potential, as well as localization and targeting by the adaptive immune system in NOD mice in a way that is associated with an increased T1D prevalence. Moreover, the observation of these phenotypes to be independent of T1D precocity suggests that other factors are at play in dictating diabetes establishment.

### Dietary emulsifier consumption induced epithelium damage and low-grade intestinal inflammation in NOD mice

**Fig. 5:**
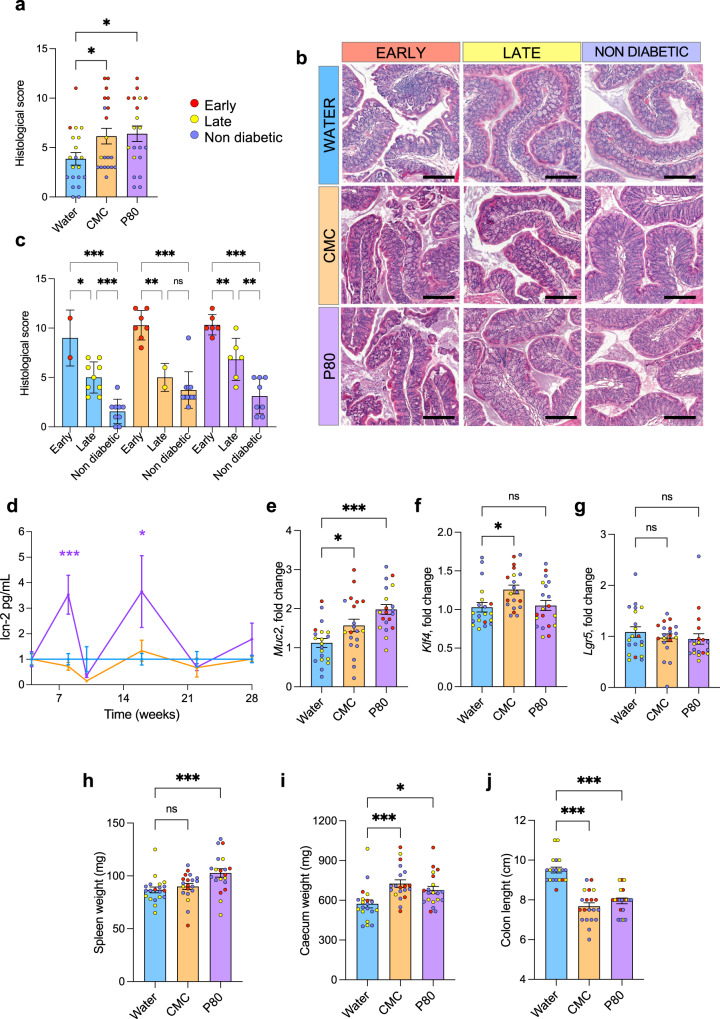
Dietary emulsifiers consumption induces low-grade intestinal inflammation in NOD mice. NOD mice were exposed to CMC or P80 (1%) in the drinking water for 28 weeks and diabetes development was evaluated weekly through blood glucose measurements. **a** and **b** Colon histological score was determined and plotted according to treatment groups (**a**) or treatment subdivided by T1D status (**b**). **c** Representative images of HE-stained transversal colonic sections. Scale bar: 200 μm. **d** Fecal level of pro-inflammatory host-derived lipocalin-2 over time, with data being normalized to water-treated mice and to week 4 (pre-treatment) timepoint. **e** and **g** Colonic expression fold change of Muc2 (**e**), Klf4 (**f**), and Lgr5 (**g**). **h** and **j** Spleen weight (**h**), cecum weight (**i**), and colon length (**j**) were measured at euthanasia. Data are the means ± s.e.m., and points represent individual mice (*N* = 20). Significance are indicated as *p ≤ 0,05, **p ≤ 0,01; ***p ≤ 0,001; n.s. indicates nonsignificant.

Dietary emulsifiers induce low-grade inflammation which drives microbiota-dependent deleterious consequences on host health^14^. To investigate to which extent this also applies to NOD mice in the context of T1D development, and the potential relationship between intestinal inflammation and T1D precocity, we next quantified epithelium inflammation via colon histological scoring. Both CMC- and P80-treated mice harbored a significant increase in colon histological scoring (Fig. 5a, b). Moreover, a strong association was observed between intestinal inflammation level and T1D precocity, with the highest score being harbored by early diabetic mice in all three experimental groups (Fig. 5b, c). We further investigated intestinal inflammation longitudinally, through the use of the fecal lipocalin-2 (Lcn2) marker (Lcn2)^29^, revealing that P80 treatment was associated with increased Lcn2 at week 8 and 16 (Fig. 5d), suggesting a transient and unstable impact of P80 emulsifier on intestinal inflammation. To further understand the impact of emulsifiers on the intestine, we next performed qRT-PCR-based quantification of the expression of colonic genes involved in epithelial homeostasis. Such an approach revealed an increased expression of Muc2 (main intestinal mucin contributing to mucus barrier formation) and Klf4 (a zinc finger transcription factor involved in Goblet cells homeostasis) in CMC- and P80-treated mice compared to water-treated mice (Fig. 5e, f), suggesting a host-compensatory response through mucus production in facing emulsifier-induced alteration in barrier-microbiota homeostasis. This response was not associated with altered colonic expression of Lgr5 following CMC or P80 treatment, suggesting that emulsifier exposure had not impacted epithelium proliferation status (Fig. 5g).

In accord with their promotion of histologically evident chronic intestinal inflammation, dietary emulsifier consumption also influenced gross morphological changes, including increased spleen weight in P80-treated mice (Fig. 5h), increased cecum weight (Fig. 5i) as well as colon shortening (Fig. 5j) in both CMC- and P80-treated mice. Such morphological changes were not associated with the earliness of T1D development, as presented in Supplementary Fig. 3b–d. Interestingly, such intestinal inflammation was observed to be distinct between CMC and P80, with a differential effect on spleen weight and lipocalin-2 levels, once again highlighting the specificity of each emulsifier on the host response. Of note, both CMC- and P80-treated mice presented a significant increase in body weight over time, which was mostly driven by diabetic mice (Supplementary Fig. 4). Hence, dietary emulsifier consumption detrimentally impacted the intestinal compartment by inducing low-grade inflammation. Importantly, the increased histological score was associated with early T1D development in NOD mice, irrespective of their experimental treatment.

### Gut microbiota functional assessment enables partial T1D status prediction

**Fig. 6:**
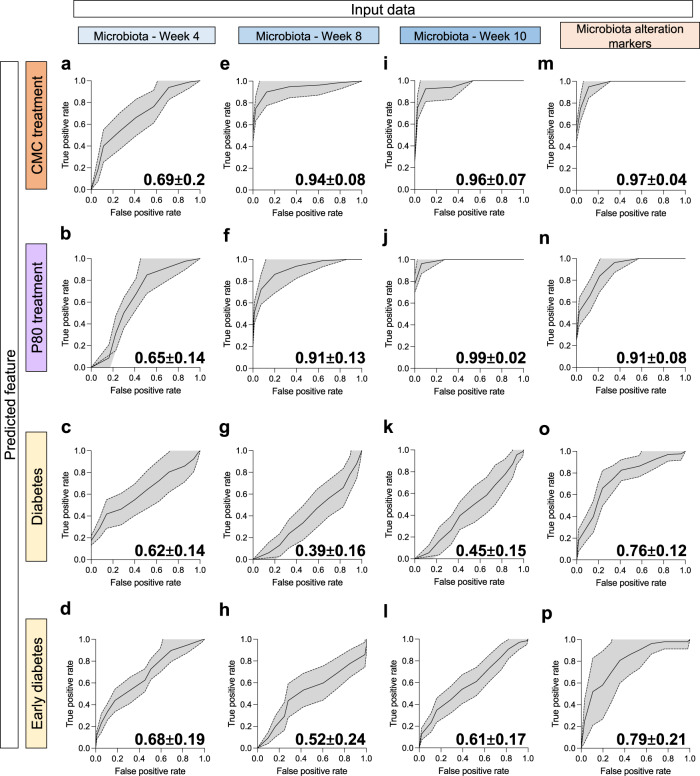
Microbiota composition and function enable treatment and early diabetes status prediction in NOD mice. NOD mice were exposed to CMC or P80 (1%) in the drinking water for 28 weeks and diabetes development was evaluated weekly through blood glucose measurements. ROC curves-based prediction of CMC treatment (outcome CMC or WATER, **a**, **e**, **i**, **m**), P80 treatment (outcome P80 or WATER, **b**, **f**, **j**, **m**), diabetic status (outcome TRUE or FALSE, **c**, **g**, **k**, **o**) or early diabetic status (outcome EARLY or LATE-OR-NO, **d**, **h**, **l**, **p**). Prediction was based on the rarefied abundance of microbiota members identified at the species level at week 4 (**a–d**), at week 8 (**e–h**), at week 10 (**i–l**), or quantitative microbiota alteration markers (fecal levels of flagellin, LPS, TLR2 ligands at weeks 8 and 10, distance from intestinal epithelial cells, week 10 fecal proportion of IgA-coated bacteria, and flagellin/LPS-specific antibodies levels at week 8 of age and euthanasia, **m–p**). Curves are mean ± s.d. of curve coordinates obtained following 20 predictive iterations with independent training/validation cohort sampling. Mean area under the curve ± s.d. is indicated on the graph.

Data presented above suggest that emulsifier consumption drove chronic intestinal low-grade inflammation, microbiota alteration, and accelerated T1D development phenotype. However, when analyzed individually, we did not observe any correlation between microbiota-associated phenotypes and T1D precocity. Hence, to assess whether integrated microbiota compositional and functional analysis could predict T1D diabetes development, we used receiver operating characteristic (ROC) curves to predict outcomes based on microbiota compositional assessment after 0, 4, and 6 weeks of treatment, as well as based on microbiota functional features (including fecal levels of flagellin, LPS, TLR2 ligands at weeks 8 and 10 of age, microbiota-IEC distance, week 10 fecal IgA coated bacteria, and flagellin/LPS specific antibodies circulating levels at week 8 of age and euthanasia). As expected, microbiota compositional data at week 4 of age (prior treatment) did not enable the prediction of future treatment nor future diabetes status and precocity (Fig. 6a–d). Microbiota compositional data at week 8 and 10 of age (after 4 and 6 weeks of treatment, respectively) were sufficient to predict treatment (Fig. 5e, f, i, j), but not diabetic status nor earliness of diabetes development (Fig. 5g, h, k, l). More importantly, markers of microbiota alteration enabled accurate treatment prediction (Fig. 5m, n), and, furthermore, increased prediction success of diabetic status (Fig. 5o) as well as earliness of diabetes development (Fig. 5p). Because CMC mice presented accelerated T1D rather than increase T1D prevalence, we performed a similar approach using a model allowing prediction of quantitative outcome (earliness of T1D development). Such an approach was not able to provide a successful prediction of the age at diagnosis (data not shown). Altogether, these observations suggest that the severity of emulsifier-induced microbiota alterations is only partly associated with diabetes status and T1D precocity, suggesting that complex interactions between the host, dietary factors, and the intestinal microbiota promote T1D.

## Discussion

The enormous burden posed by T1D on quality of life and healthcare economics compels a scrutinized understanding of the modifiable factors that drive disease development. However, despite substantial efforts using preclinical disease models and human epidemiological approaches, environmental factors that impact T1D development remain very unclear. By playing a central role in regulating both immune and metabolic pathways, the intestinal microbiota, and in particular its modulation by dietary factors, has been implicated in T1D development, with both protective^30–33^ and deleterious effects^34–36^. Observations by ourselves and others that consumption of dietary emulsifiers triggers microbiota alterations in terms of composition and encroachment leading to detrimental health consequences for the host^14,15,37,38^, prompted us to investigate whether dietary emulsifier consumption might influence T1D development.

Using the NOD mouse model, we demonstrated that CMC consumption significantly accelerated T1D development. Such diabetic precocity is associated with increased serum levels of insulin autoantibody, a marker of T1D early development. We also observed the stark impacts of dietary emulsifiers on microbiota composition and localization in a way that is associated with the promotion of low-grade intestinal inflammation, analogous to previous reports in other mouse models^14^. These altered phenotypes did not closely correlate with diabetes status or diabetes onset rapidity. Rather, we observed a strong association between histologically assessed intestinal inflammation and T1D precocity. Furthermore, machine learning assessment highlighted that the severity of emulsifier-induced microbiota alterations is partly associated with diabetes status and T1D precocity, suggesting that complex interactions occur between the host, dietary factors, and the intestinal microbiota to promote T1D. Hence, further work is needed to identify microbiota-dependent and independent mechanisms underlying the effect of emulsifier consumption on T1D development.

Potential mechanisms by which altered microbiota might promote T1D are numerous, with previous mechanistic studies bringing to light the role of microbiota-derived SCFA^30,32,39^, TLR receptors^23^, enterovirus^40^, and host-derived anti-microbial peptides^41,42^ in mediating interactions between gut microbiota and T1D development. Such mechanistic gaps notwithstanding, our current study provide mechanistic clues on previously reported correlations between microbiota encroachment and dysglycemia in humans^43^. Indeed, we previously observed that the extent of microbiota encroachment correlates with the severity of type 2 diabetes (T2D) in humans, with the cause/consequence relationship remaining unclear. Indeed, while microbiota encroachment could be a central feature for the promotion of chronic low-grade intestinal inflammation and downstream development of T2D, increased glycemia could also result in glucose leak, forming a glucose gradient from the intestinal epithelium towards the intestinal lumen ultimately attracting bacteria towards the IECs and inducing encroachment. However, the absence of correlation between glycemia and microbiota encroachment observed in the present study challenges the glucose leak hypothesis and, rather suggests that microbiota encroachment is a cause rather than a consequence of dysregulated glucose homeostasis.

This current study brings new insight regarding the effect of dietary emulsifiers on the development of T1D, of major importance considering the rapid increase of both T1D incidence and the broad use of dietary emulsifiers in processed food production. While we identified the deleterious effect of such additives on T1D development, ultra-processed foods are characterized by the use of numerous additives, including emulsifiers, as well as other nutritional characteristics including high amounts of sugars^44^ and low amounts of fibers^30^ which could altogether act synergistically in T1D development, as well as other autoimmune diseases. Moreover, the NOD mice model used in the current study has a major limitation in its relatively rapid and penetrant T1D development, with diagnosis occurring between the ages of 10- to 30-week-old while in humans, T1D development, despite being mainly diagnosed in children, takes years to develop, during which emulsifiers are chronically consumed in a way that could deleteriously act on T1D development.

To conclude, we herein report that emulsifier consumption accelerates T1D development. The severity of emulsifier-induced microbiota disruption had partial power to predict subsequent disease development, suggesting that complex interactions occur between the host, dietary factors, and the intestinal microbiota to promote T1D. Further work appears needed to identify the exact mechanisms by which dietary emulsifiers influence T1D development in NOD mice, as this might open therapeutic approaches to beneficially impact microbiota in a manner to prevent T1D development.

## Methods

### Mice

Four-week-old female NOD/ShiLtJ mice were purchased from Jackson Laboratories. Mice were housed in cages of 5 mice and were kept under a 12 h light/dark cycle and had free access to standard chow diet and water (water-treated group, 4 cages) or water with 1% CMC (CMC-treated group, 4 cages) or water with 1% P80 (P80-treated group, 4 cages). Cages from all groups were changed every other week. Body weight was measured and feces were collected every other week. From week 8 of age, glycemia was checked every 2 weeks and every week from week 12 of age. Serum was collected at week 8 of age. Type one diabetes status was declared after 2 glycemia measurements above 200 mg dL^−1^ 72 h apart. Upon diabetes diagnosis, or after 28 weeks of age, mice were euthanized by isoflurane and cervical dislocation. Colon length, colon weight, spleen weight, and adipose weight were measured and organs were collected for downstream analysis. Animal welfare and experimental protocols followed the ARRIVE guidelines (Animal Research: Reporting of In Vivo Experiments). All procedures involving animals were approved by the French *Ministère de la l’enseignement supérieur, de la recherche et de l’innovation*, APAFIS#24788-2019102806256593 v8.

### Quantification of serum insulin auto-antibody by ELISA

Insulin-specific serum IgG levels were quantified by ELISA. Microtiter plates were coated overnight with purified mouse insulin (100 ng per well). Serum samples diluted 1:40 were then applied. After incubation and washing, wells were incubated with HRP-linked anti-mouse IgG (1:1000, SouthernBiotech, 1015-05). Quantification was performed using the colorimetric peroxidase substrate tetramethylbenzidine and optical density was read at 450 nm (Versamax microplate reader). Data are reported as optical density corrected by subtracting background (determined by readings in samples lacking serum).

### Quantification of serum flagellin or LPS-specific IgG by ELISA

Flagellin and LPS-specific serum IgG levels were quantified by ELISA. Microtiter plates were coated overnight with purified flagellin from *Salmonella* Typhimurium (Sigma, 100 ng per well) or LPS (from *E. coli* 0128: B12, Sigma, 2 μg per well) diluted in carbonate–bicarbonate buffer. Serum samples diluted 1:200 were then applied. After incubation and washing, wells were incubated with HRP-linked anti-mouse IgG (1:1000, SouthernBiotech, 1015-05). Quantification was performed using the colorimetric peroxidase substrate tetramethylbenzidine and optical density was read at 450 nm (Versamax microplate reader). Data are reported as optical density corrected by subtracting background (determined by readings in samples lacking serum).

### Quantification of fecal LCN2 by ELISA

For quantification of fecal LCN2 by ELISA, frozen fecal samples were reconstituted in PBS containing 0.1%Tween 20 to a final concentration of 100 mg ml^−1^ and vortexed for 20 min to produce a homogenous fecal suspension^29^. These samples were then centrifuged for 10 min at 14,000×*g* and 4 °C. Clear supernatants were collected and stored at −20 °C until analysis. LCN2 levels were estimated in the supernatants using a Duoset murine LCN2 ELISA kit (R&D Systems, Minneapolis, MN, USA) using the colorimetric peroxidase substrate tetramethylbenzidine, and optical density was read at 450 nm (Versamax microplate reader).

### Insulinitis scoring

Following euthanasia, pancreases were harvested and fixed in 4% paraformaldehyde, embedded in paraffin, sectioned, and stained with hematoxylin and eosin. Insulitis was evaluated blindly and as previously described^24^. Briefly, all islets on the slide were analyzed and a score between 0 and 3 was given to each. 0 = no insulitis, 1 = peri-insulitis, 2 = infiltration < 50% of the islet and 3 = infiltration > 50% of the islet.

### Hematoxylin and eosin staining and histopathologic analysis

Following euthanasia, colons (proximal colon, 2 first cm from the cecum) were placed in Carnoy’s fixative solution (60% methanol, 30% chloroform, 10% glacial acetic acid). Tissues were then washed in methanol 2 × 30 min, ethanol 2 × 15 min, ethanol/xylene (1:1) 15 min, and xylene 2 × 15 min, followed by embedding in paraffin with a vertical orientation. Tissues were sectioned at 5-μm thickness and stained with hematoxylin & eosin (H&E) using standard protocols. H&E-stained slides were assigned four scores based on the degree of epithelial damage and inflammatory infiltrate in the mucosa, submucosa, and muscularis/serosa. Each of the four scores was multiplied by 1 if the change was focal, 2 if it was patchy, and 3 if it was diffuse, as previously described^29^. The four individual scores per colon were added, resulting in a total scoring range of 0–36 per mouse.

### Microbiota analysis by 16S rRNA gene sequencing

16S rRNA gene amplification and sequencing were done using the Illumina MiSeq technology following the protocol of Earth Microbiome Project with their modifications to the MOBIO PowerSoil DNA Isolation Kit procedure for extracting DNA (www.earthmicrobiome.org/emp-standard-protocols). Bulk DNA were extracted from frozen extruded feces using a PowerSoil-htp kit from MoBio Laboratories (Carlsbad, CA, USA) with mechanical disruption (bead-beating). The 16S rRNA genes, region V4, were PCR amplified from each sample using a composite forward primer and a reverse primer containing a unique 12-base barcode, designed using the Golay error-correcting scheme, which was used to tag PCR products from respective samples^45^. We used the forward primer 515F 5’- *AATGATACGGCGACCACCGAGATCTACACGCT*XXXXXXXXXXXX**TATGGTAATT*GT***GTGYCAGCMGCCGCGGTAA-3’: the italicized sequence is the 5’ Illumina adapter, the 12 X sequence is the golay barcode, the bold sequence is the primer pad, the italicized and bold sequence is the primer linker and the underlined sequence is the conserved bacterial primer 515F. The reverse primer 806R used was 5’-*CAAGCAGAAGACGGCATACGAGAT***AGTCAGCCAG*CC*** GGACTACNVGGGTWTCTAAT-3’: the italicized sequence is the 3’ reverse complement sequence of Illumina adapter, the bold sequence is the primer pad, the italicized and bold sequence is the primer linker and the underlined sequence is the conserved bacterial primer 806R. PCR reactions consisted of Hot Master PCR mix (Quantabio, Beverly, MA, USA), 0.2 μM of each primer, 10–100 ng template, and reaction conditions were 3 min at 95 °C, followed by 30 cycles of 45 s at 95 °C, 60 s at 50 °C, and 90 s at 72 °C on a Biorad thermocycler. Products were then visualized by gel electrophoresis and quantified using Quant-iT PicoGreen dsDNA assay (Clariostar Fluorescence Spectrophotometer). A master DNA pool was generated in equimolar ratios, subsequently purified with Ampure magnetic purification beads (Agencourt, Brea, CA, USA) and sequenced using an Illumina MiSeq sequencer (paired-end reads, 2 × 250 bp) at the Genom’IC platform (INSERM U1016, Paris, France).

### 16S rRNA gene sequence analysis

16S rRNA sequences were analyzed using QIIME2—version 2019^46^. Sequences were demultiplexed and quality filtered using the Dada2 method^47^ with QIIME2 default parameters in order to detect and correct Illumina amplicon sequence data, and a table of QIIME2 artifact was generated using the following dada2 command: qiime dada2 denoise-paired --i-demultiplexed-seqs demux.qza --p-trim-left-f 0 --p-trim-left-r 0 --p-trunc-len-f 180 --p-trunc-len-r 180 --o-representative-sequences rep-seqs-dada2.qza --o-table table-dada2.qza --o-denoising-stats stats-dada2.qza --p-n-threads 6. A tree was next generated, using the align-to-tree- mafft-fasttree command, for phylogenetic diversity analyses, and alpha and beta diversity analyses were computed using the core-metrics-phylogenetic command. Principal coordinate analysis (PCoA) plots were used to assess the variation between the experimental group (beta diversity). For taxonomy analysis, features were assigned to operational taxonomic units (OTUs) with a 99% threshold of pairwise identity to the SILVA reference database^48^.

### Fecal flagellin, LPS, and TLR2 ligands load quantification

Levels of fecal bioactive flagellin, LPS, and TLR2 ligands were quantified as previously described^49^ using human embryonic kidney (HEK)-Blue-mTLR5, HEK-Blue-mTLR4, and HEK-Blue-mTLR2 cells, respectively (Invivogen, San Diego, CA). We resuspended fecal material in PBS to a final concentration of 100 mg mL^−1^ and homogenized for 15 minutes using a vortex. We then centrifuged the samples at 8000×*g* for 15 min and serially diluted the resulting supernatant and applied it to mammalian cells. Purified *Escherichia coli* flagellin, LPS (Sigma, St. Louis, MO, USA), and TLR2 ligands were used for standard curve determination using HEK-Blue-mTLR5, HEK-Blue-mTLR4 and HEK-Blue-mTLR2 cells, respectively. After 24 h of stimulation, we applied cell culture supernatant to QUANTI-Blue medium (Invivogen, San Diego, CA, USA) and measured alkaline phosphatase activity at 620 nm after 30 min.

### Immunostaining of mucins and localization of bacteria by fluorescent in situ hybridization

Mucus immunostaining was paired with fluorescent in situ hybridization (FISH), as previously described^14,50^, in order to analyze bacteria localization at the surface of the intestinal mucosa. In brief, colonic tissues (proximal colon, second cm from the cecum) containing fecal material were placed in methanol-Carnoy’s fixative solution (60% methanol, 30% chloroform, 10% glacial acetic acid) for a minimum of 3 h at room temperature. Tissues were then washed in methanol 2 × 30 min, ethanol 2 × 15 min, ethanol/xylene (1:1) 15 min, and xylene 2 × 15 min, followed by embedding in paraffin with a vertical orientation. Five-μm sections were cut and dewaxed by preheating at 60 °C for 10 min, followed by bathing in xylene at 60 °C for 10 min, xylene at room temperature for 10 min, and 99.5% ethanol for 10 min. The hybridization step was performed at 50 °C overnight with an EUB338 probe (59-GCTGCCTCCCGTAGGAGT-39, with a 59 Alexa 647 label) diluted to a final concentration of 10 mg mL^−1^ in hybridization buffer (20 mM Tris–HCl, pH 7.4, 0.9 M NaCl, 0.1% SDS, 20% formamide). After washing for 10 min in wash buffer (20 mM Tris–HCl, pH 7.4, 0.9 M NaCl) and 3×10 min in PBS, a PAP pen (Sigma, St. Louis, MO, USA) was used to mark around the section and block solution (5% FBS in PBS) was added for 30 min at 4 °C. Mucin-2 primary antibody (rabbit H-300, [C3], C-term, Genetex, GTX100664) was diluted to 1:100 in block solution and applied overnight at 4 °C. After washing 3 × 10 min in PBS, block solution containing anti-rabbit Alexa 488 secondary antibody diluted to 1:300, PhalloidinTetramethylrhodamine B isothiocyanate (Sigma-Aldrich) at 1 mg ml^−1^ and Hoechst 33258 (Sigma-Aldrich) at 10 mg ml^−1^ was applied to the section for 2 h. After washing 3 × 10 min in PBS slides were mounted using Prolong anti-fade mounting media (Life Technologies) and kept in the dark at 4 °C. Observations and measurement of the distance between bacteria and epithelial cell monolayer were performed with a Spinning Disk IXplore using the Olympus cellSens imaging software 421 (V2.3) at a frame size of 2048 × 2048 with 16-bit depth. A 405 nm laser was used to excite the 422 Hoechst stain (epithelial DNA), 488 nm for Alexa Fluor 488 (mucus), 488 nm for Phalloidin (actin), 423 and 640 nm for Alexa Fluor 647 (bacteria). Samples were imaged with a ×20 objective.

### Quantification of fecal IgA-coated bacteria and bacterial load

IgA-coated bacteria were quantified as previously described^28^. In brief, frozen fecal samples were thoroughly homogenized in PBS to a final concentration of 20 mg/ml. Fecal suspensions were filtered through a 40-μm sterile nylon mesh, then centrifuged at 50×*g*, for 15 min at 4 °C. 200 μl of supernatant was then washed with 1 ml PBS and centrifuged at 8000×*g*, for 5 min at 4 °C. Resulting bacterial pellets were resuspended in 100 μl blocking buffer (staining buffer containing 20% Normal Rat Serum) and incubated for 20 min on ice before being stained with 100 μl of staining buffer containing PE-conjugated Anti-Mouse IgA (1:12.5; eBioscience, 12-4204-82) for 30 min on ice, in the dark. Following two washes with staining buffer, pellets were resuspended in 200 μl of FACS buffer (PBS, 1% Normal Rat Serum). Data acquisition was performed on a Beckman Coulter Gallios flow cytometer. For each sample, 50,000 events were recorded and data was analyzed using FlowJo software v.10.8.2. Bacterial load was quantified by gating bacterial population based on size and granularity.

### Colonic RNA extraction and q-RT-PCR analysis

Distal colon was collected during euthanasia and placed in RNAlater. Total mRNAs were isolated from colonic tissues using TRIzol (Invitrogen, Carlsbad, CA) according to the manufacturer’s instructions and as previously described^29^. Quantitative RT-PCR was performed using the Qiagen kit QuantiFast® SYBR® Green RT-PCR in a LigthCycler® 480 instrument (Roche Molecular Systems, Inc.) with specific mouse oligonucleotides (Supplementary Table 1). Gene expressions are presented as relative values using the Ct approach with the Gapdh housekeeping gene.

### Treatment and diabetic status prediction based on microbiota composition

The association between microbiota composition and treatment or diabetic status was assessed using prediction based on microbiota composition or pro-inflammatory potential data of either CMC treatment (outcome CMC or WATER, Fig. 6a, e, i, m), P80 treatment (outcome P80 or WATER, Fig. 6b, f, j, m), diabetic status (outcome TRUE or FALSE, Fig. 6c, g, k, o) or early diabetic status (outcome EARLY or LATE-OR-NO, Fig. 6d, h, l, p). Receiver operating characteristic (ROC) curves were calculated (R version 4.1.2, randomForest 4.7-1.1 package, ROCR package) using training data set and validation data set containing randomly affected 80% and 20% of mice, respectively. Data set contained relative abundance data for microbiota members identified at the species level at week 4 (Fig. 6a–d), at week 8 (Fig. 6e–h), at week 10 (Fig. 6i–l) or quantitative microbiota functional features (fecal levels of flagellin, LPS, TLR2 ligands at weeks 8 and 10, distance from intestinal epithelial cells, week 10 fecal proportion of IgA-coated bacteria, and flagellin/LPS specific antibodies levels at week 8 of age and euthanasia, Fig. 6m–p). ROC calculation was repeated 20 times with random sampling of the training and validation data and area under curve (AUC) measurement for each iteration. Mean AUC and standard deviation are presented for each graph.

### Identification of microbiota members significantly altered in their relative abundance

Microbiota members presenting significant changes in abundance after 6 weeks of CMC or P80 treatment were identified using MaAsLin2 (Microbiome Multivariable Associations with Linear Models, version 2)^51^. MaAsLin2 analysis (R version 4.1.2, Maaslin2 version 1.12.0 package) was conducted using relative abundance data for microbiota members identified at the species level at week 10. Microbiota members were reported as significantly altered in their relative abundance if corrected *p*-value < 0.05. Obtained data were reported as a Log2 fold change in relative abundance compared to the mean abundance observed in the Water-treated group.

### Statistical analysis

Significance was determined using a log-rank Mandel–Cox test in Fig. 1a. When normality and homoscedasticity postulates were valid, significance was tested using one-way group analysis of variance (ANOVA) with Sidak’s multiple comparisons test (Figs. 1c–e; 3j; 4c, d, g; 5a, b, e, j; Supplementary Figs. 2, 3). Significance of data that did not respect normality and homoscedasticity postulates was tested using Kruskal–Wallis corrected for multiple comparisons with a Dunn’s test (Figs. 1b; 4a, f; 5d, f–i) or Brown–Forsythe and Welch ANOVA corrected for multiple comparisons with a Dunnett test (Fig. 4e), respectively. Significance of longitudinally measured data was assessed using two-way ANOVA corrected for multiple comparisons with Sidak’s test (Figs. 2e, f; 3a–i; Supplementary Fig. 4). Clustering significance in Fig. 2a–d was determined using Permutational multivariate analysis of variance (PERMANOVA) and corrected *p*-values are indicated on plots. Differences were noted as significant *p ≤ 0,05; **p ≤ 0,01; ***p ≤ 0,001; ****p ≤ 0,0001; n.s. indicates nonsignificant.

### Reporting summary

Further information on research design is available in the Nature Research Reporting Summary linked to this article.

### Supplementary information

Supplemental material (figures and table)

Reporting summary

## Data Availability

Unprocessed sequencing data are deposited in the European Nucleotide Archive under accession number PRJEB67708.
