# Supplementary material for: Dietary emulsifier consumption accelerates type 1 diabetes development in NOD mice
Source: NPJ Biofilms Microbiomes. 2024 Jan 6;10:1. doi: 10.1038/s41522-023-00475-4 (PMC10770373; doi:10.1038/s41522-023-00475-4)
Supplement: Supplementary file 1 — Supplemental material (figures and table) [file 41522_2023_475_MOESM1_ESM.pdf]

a

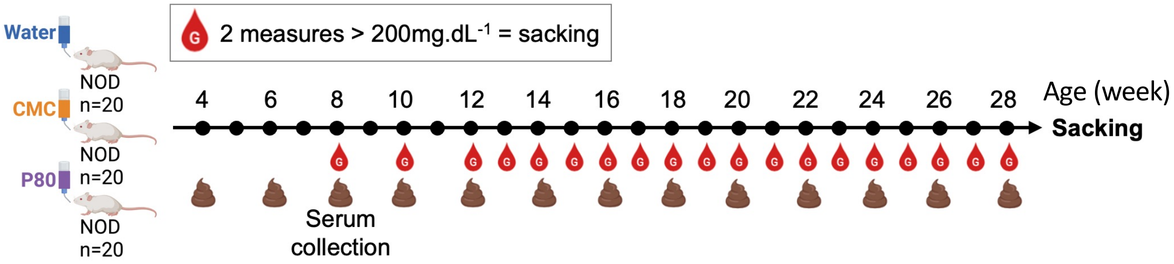

b

Group	Age at diagnosis (weeks)
Early	[0-19]
Late	[20-28]
Non diabetic	X

Supplementary Figure 1. Schematic representation of the experimental design used. NOD mice (N = 20 mice per group) were exposed to CMC or P80 (1%) in the drinking water for 28 weeks and diabetes development was evaluated weekly through blood glucose measurements. (**a**) Experimental design. (**b**) Range of age at diagnosis used to define early *vs.* late diabetic mice throughout the study.

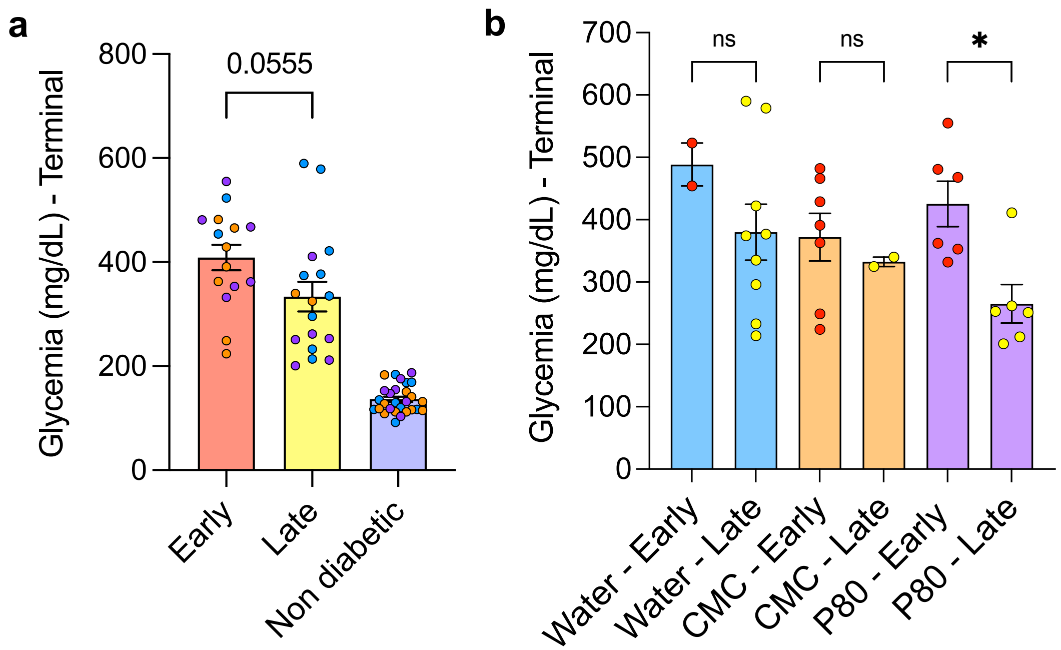

Supplementary Figure 2. Glycemia at diagnosis based on diabetes status. NOD mice (N = 20 mice per group) were exposed to CMC or P80 (1%) in the drinking water for 28 weeks and diabetes development was evaluated weekly through blood glucose measurements. **a-b**. Glycemia at diagnosis, presented based on diabetes status (**a**), or diabetes status within treatment groups (**b**). Data are the means ± s.e.m and points represent individual mice. Significance is indicated as *p ≤ 0,05; n.s. indicates nonsignificant.

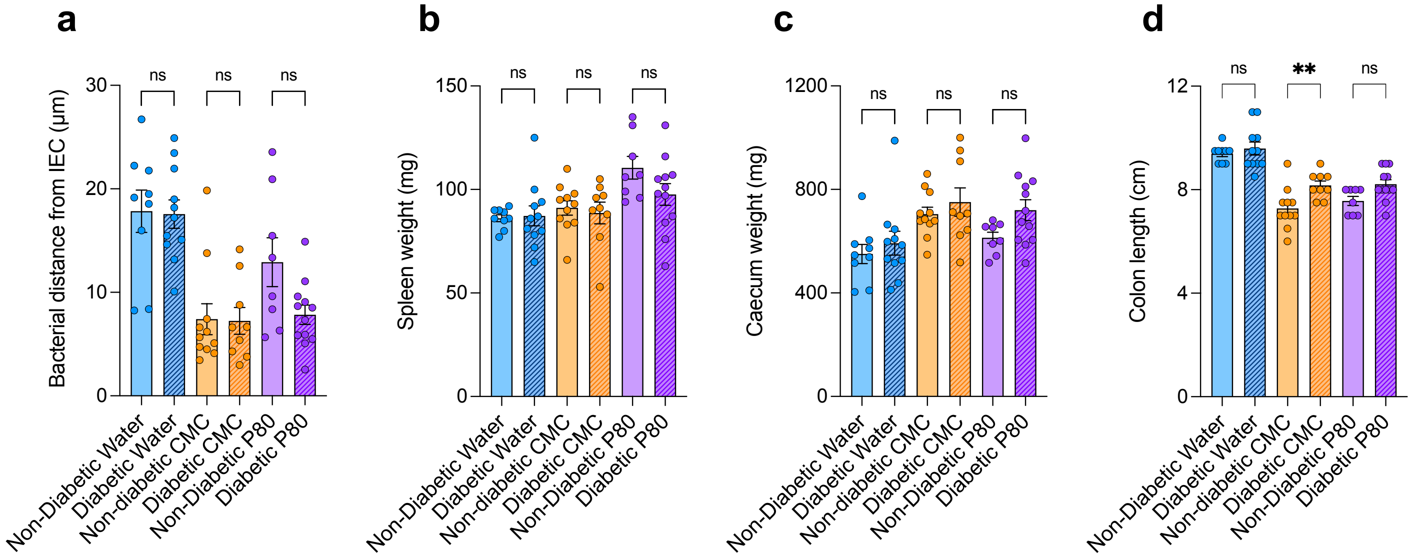

Supplementary Figure 3. Dietary emulsifiers consumption induces low-grade intestinal inflammation in both diabetic and non-diabetic NOD mice. NOD mice were exposed to CMC or P80 (1%) in the drinking water for 28 weeks and diabetes development was evaluated weekly through blood glucose measurements. **a**. Distances of closest bacteria to intestinal epithelial cells (IECs) over five high-powered fields per mouse. **b-d** Spleen weight (**b**), caecum weight (**c**) and colon length (**d**) measured at euthanasia. The data presented here are the same as panel **4a**, **5g**, **5h** and **5i** but after subdivision by T1D status. Data are the means ± s.e.m and points represent individual mice. Significance is indicated as *p ≤ 0,05; ***p ≤ 0,001; n.s. indicates nonsignificant.

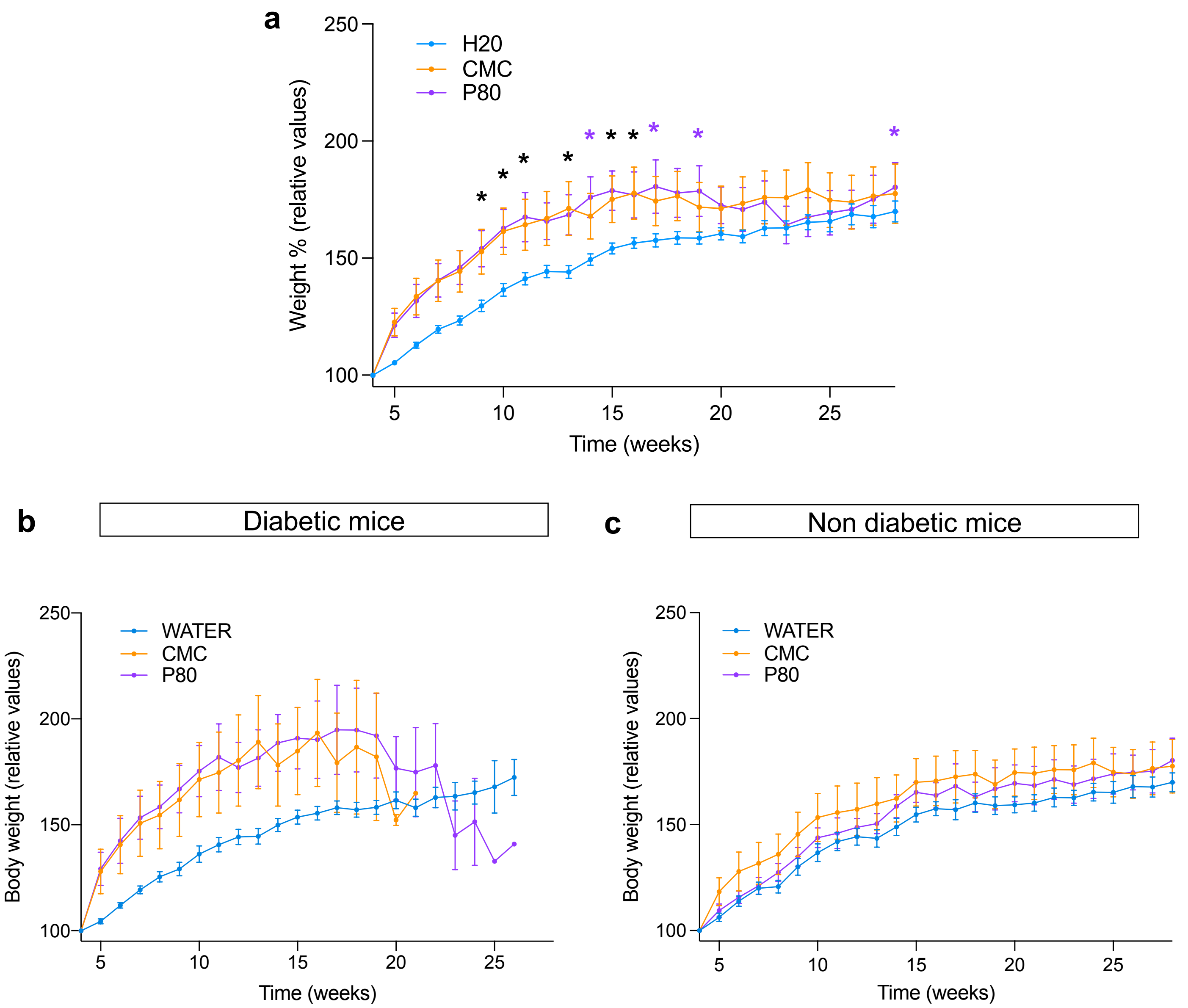

Supplementary Figure 4. Dietary emulsifiers consumption induces increase in body weight over time in NOD mice. NOD mice were exposed to CMC or P80 (1%) in the drinking water for 28 weeks and diabetes development was evaluated weekly through blood glucose measurements. Body weight evolution are expressed as percentage compared to week 4, defined as 100%. Panel **a** present all the animals per group, while panel **b** represents only diabetic mice and panel **c** represents only non-diabetic free mice. Data are mean +/- s.e.m (N=20), significance is indicated as *p ≤ 0,05.

Gene	Sequence
Klf4	*F - AGAACAGCCACCCACACTTG*
	R - CCCTGTGTGTTTGCGGTAGT
Lgr5	*F - GTGGACTGCTCGGACCTG*
	R - GCTGACTGATGTTGTTCATACTGAG
Muc2	*F - AAACTGCTCTCTGGACTGCC*
	R - TTGGTTGGTGTGCTGAGTGT

Supplementary table 1. Primers sequences.
